# Supplementary material for: Are carbon emissions trading and green financial instruments synergistic? -Comprehensive quantitative research based on content analysis
Source: PLoS One. 2024 Mar 7;19(3):e0298601. doi: 10.1371/journal.pone.0298601 (PMC10919601; doi:10.1371/journal.pone.0298601)
Supplement: S1 Data — (ZIP) [file pone.0298601.s001.zip › Data/地级市Z_F工作报告.zip!/╜¡╦╒/│ú╓▌╩╨/└┤╫╘╣1⁄2╓┌║┼í╛┬φ┐╦╩2╛▌═°í┐_2008.docx]

各位代表：

　　现在，我代表常州市人民政府，向大会作工作报告，请予审议，并请市政协各位委员和其他列席人员提出意见。

　　一、过去五年工作的回顾总结

　　本届政府组成以来，在中共常州市委的正确领导下，在市人大和市政协的监督支持下，紧紧依靠全市人民，围绕实现全面小康总目标，解放思想，锐意进取，创新创造，克难求进，圆满完成了市第十三届人大各次会议确定的目标任务。

　　（一）经济建设蒸蒸日上，综合实力明显增强。2007年，预计全市实现地区生产总值1880亿元，是2002年的2.5倍，按可比价年均增长15.2%，经济总量由全省第六位上升到第五位，户籍人均水平超过7000美元；财政总收入414亿元，地方一般预算收入158亿元，分别是2002年的4倍、3.4倍。全社会固定资产投资五年累计完成近4000亿元，是前五年的4.5倍。农业产业化水平不断提升，高效农业种养面积已占总种养面积的1/3，水稻单产、机械化种植率连续五年位居全省第一。工业经济优化发展，去年规模以上工业总产值达到4250亿元，是2002年的3.6倍，高新技术产业产值所占比重达到42.1%，五年提高了12.5个百分点。服务业发展速度加快，去年增加值可比价增幅达到16%，占三次产业的比重达到37%，旅游总收入是2002年的3.1倍。

　　（二）人民生活不断改善，提前实现全面小康。城乡居民收入持续增长，预计去年城镇居民人均可支配收入19100元，农民人均纯收入8960元，分别比2002年增长了92.3%、74.4%；居民人民币储蓄存款余额1092亿元，增长了1.18倍；社会消费品零售总额606亿元，增长了1.4倍，增速居全省前列。社会保障体系不断完善，城镇养老、医疗、失业三大保险综合覆盖率达到97%以上，慈善基金突破12亿元。人民群众更多享受到改革发展成果，公交优先、公园免费开放、菜市场改造、老小区整治、住房保障、茅山老区扶贫帮困等一大批实事好事先后实施。去年成为全省第三个以县为单位达到全面小康目标的省辖市。

　　（三）改革开放步伐加快，创新创业硕果累累。对外开放取得新的突破。五年累计实际到账注册外资52.4亿美元，去年达到18.3亿美元，已有46家世界500强企业落户常州；全市进出口总额累计434亿美元，其中出口318亿美元，年均分别增长26.1%和29.0%；外经营业额10.3亿美元。出口加工区和海关、国检武进办事处建成投运。各项改革有序推进。完成工业企业和经营性事业单位改革改制任务，新增境内外上市企业8家，累计达15家；全面免征农业税及其附加，积极推进农村“三大合作”改革，开展了农业保险试点工作；实施了街道、乡镇两轮行政区划调整，街道、乡镇分别减少了16.6%、42.18%；财税、金融、文化体制改革也取得了明显成效。民营经济迅猛发展。私营企业和个体工商户分别达到5.42万户和12.25万户，注册资本达880亿元；民营经济占全市经济总量、税收总额、固定资产投资的比重均超过60％。科技创新能力不断增强。依托大学、大院、大所开展产学研合作成效显著，五年新增省级以上高新技术企业460家、研发机构126家，新增中国驰名商标6件、中国名牌产品25只，专利申请、授权量分别达到12981件、6212件。

　　（四）城乡建设全面推进，各项事业协调发展。编制完成了新一轮城市总体规划。区域交通枢纽地位基本确立，高速公路通车里程达到222公里，新建改建国省干线公路331公里、农村公路2600公里，等级公路密度处于全省领先水平；京杭运河常州市区段改线工程顺利竣工，录安洲港区开工建设，常州港吞吐量超过3000万吨；常州机场与深航成功合作，去年旅客吞吐量近60万人次。城市框架进一步拉开，南北新城初具规模，城市化率达到61.0%，比2002年提高了5.2个百分点；建设了高架路、青洋路、长江路等一批骨干道路，新建、扩建城市道路近1000公里。城市功能日趋提升，实施了城乡公交一体化整合，市区范围行政村公交通达率超过90％，建成快速公交一号线；一批供水供气、垃圾污水处理等市政公用工程建成投运。城乡环境面貌显著改善，大面积实施绿化造林，市区五年新增绿地5291.9公顷，建成区绿化覆盖率达到41.6%；开展了主要道路市容环境和市区水环境综合整治，全面落实13项城市长效管理制度；城市防洪体系建设取得成效，水利工作得到明显加强；新农村建设积极推进，农村环境综合整治全面实施。社会事业蓬勃发展。教育现代化整体推进，各类教育均衡协调发展，科教城建设取得重大成绩。卫生基础设施建设得到加强，医疗水平不断提高。取得了省第十七届运动会的承办权。文化事业繁荣发展，精品生产成绩突出，博物馆、规划馆、天宁宝塔等相继建成。“平安常州”建设成效显著，社会秩序稳定良好。人防、国防和民兵预备役建设继续推进，连续四届荣获“全国双拥模范城”称号。外事侨务、对台事务、审计统计、海关国检、边防海事、邮电供电、档案地方志、防震减灾、民族宗教、老龄、妇女儿童、人口和计划生育、残疾人事业、关心下一代等工作都取得了新成绩。

　　（五）政府效能逐步提高，发展环境得到优化。按照“诚信、透明、简化、明确、高效”的要求，简化办事程序，提高办事效率。行政审批大幅减少，审批总事项由927项减少为416项，减幅达到55.1%，许可类、非许可类行政审批工作日分别压缩了35%和34%，取消、降低了221个收费项目。全面推行企业登记注册告知承诺制和“宁静生产日”制度。投资发展环境明显改善，在“全国城市创新环境评价”中名列第七位。自觉接受市人大、政协和各民主党派的监督，2747件建议和提案全部办结。认真纠正行业不正之风，坚决查处大案要案，勤政廉政建设得到加强。

　　总的来说，过去的五年，是贯彻落实科学发展观，加快体制转轨、社会转型、结构调整和经济发展方式转变的五年；是创新发展理念和思路，城市化、信息化、工业化、市场化和国际化互动并进的五年；是经济持续快速健康发展和社会全面进步，不断跨上新台阶、开创新局面的五年；是全面小康建设加速向前推进，综合实力提升最快、城乡面貌变化最大、人民得到实惠最多的五年。

　　五年的发展成果来之不易，我们要倍加珍惜；五年的发展实践丰富深刻，我们要认真总结。五年的主要体会是：

　　1．发展经济是解决一切问题的基础和关键，任何时候、任何情况下，都要牢牢抓住经济建设这个中心不动摇。五年来，我们面对“非典”等特殊事件的严峻考验，全市上下万众一心，咬住科学发展不动摇，始终把发展作为第一要务，用发展的办法来解决前进道路上遇到的各种矛盾和困难，巩固和提升了稳定和谐的好局面。

　　2．坚持以人为本，实现好、维护好、发展好人民群众的根本利益，是一切工作的出发点和落脚点。发展经济最终是为了人民，“一切为了人，依靠一切人，造福所有人”是我们始终遵循的一条基本原则，也是我们解决一切问题的基本路径。只有以致富造福人民为己任，多办老百姓得益的实事好事，才能赢得人民群众的拥护和支持。

　　3．抓住牵动全局的主要工作、事关群众利益的突出问题，组织重点突破，是推进工作的有效方法。近几年来，我们先后开展了“加快有效投入年”、解决“三难一乱”等活动，实施了“八路八口绿化”和“水环境整治”等工程，有力促进了利用外资、绿化环境、交通拥堵等问题的突破。

　　4．用创新的办法破解发展中的难题，及时化解经济运行和社会活动中的矛盾和问题，是实现既定目标的重要保证。五年来，面对发展过程中遇到的项目审批、土地、资金、能源等一系列问题，我们更新发展理念，创新工作思路，善于创造融通，有效化解了各种制约因素。

　　5．营造良好氛围，建好发展平台，构建具有比较优势的投资环境，是增强区域竞争能力的有效手段。抓环境就是抓经济、抓民生、抓和谐。五年来，我们一手抓硬环境建设，一手抓软环境提升，先后出台了优化环境若干规定，提出了“放、减、控、改、帮”五字方针和“四个一”的要求，有力促进了投资环境的改善。

　　各位代表，五年来我们所取得的一切成绩，都是中共常州市委正确领导的结果，是全市人民心齐气顺、团结奋斗的结果，凝聚着全市上下的智慧和汗水，包含着方方面面的帮助和支持。在此，我代表市人民政府，向全市人民，向市人大代表、政协委员、各民主党派、工商联、各人民团体、离退休老同志、无党派及各界人士，向驻常部队指战员、武警官兵和部、省驻常单位，向在常的中外投资者、科技工作者和所有务工人员，向所有关心、支持常州建设和发展的海内外朋友们，表示衷心的感谢和崇高的敬意！

　　我们在总结成绩的同时，也清醒地看到工作中存在的问题和不足。主要表现在：经济发展和资源环境矛盾仍然十分突出，节能减排和环境保护的压力很大；产业结构调整进展比较缓慢，投入产出强度不高，自主创新能力还不强，现代服务业、高新技术产业比重仍然偏低；对外开放程度还不够，利用外资的水平还不高，外资项目中能够带动产业升级的大项目不多；全面小康建设的成果还有待进一步巩固，确保农民持续增收、建设社会主义新农村任重道远；政府自身建设还不能完全适应形势的要求，对企业和群众的服务还存在不少薄弱环节。这些问题和矛盾，在今后的工作中要切实加以解决。

　　二、今后五年的发展目标和任务

　　党的十七大为我国未来的发展描绘了新的蓝图，作为长三角苏锡常板块重要组成部分的常州，也进入了优化发展的历史新阶段。我们要认清形势，把握趋势，转变发展方式，促进经济社会又好又快发展，不失时机地向基本实现现代化迈进。

　　今后五年全市经济社会发展的主要目标是：

　　——综合实力再上新台阶。到2012年，地区生产总值超过4000亿元，地方一般预算收入超过300亿元。经济发展方式明显转变，高新技术产业产值占规模以上工业产值的比重超过50%，服务业增加值占三次产业的比重超过40%，力争形成现代装备、新能源、新材料三大优势产业，营业收入超100亿元的企业集团超过10家。全社会研发投入占地区生产总值的比重超过2.5%，万元地区生产总值综合能耗、化学需氧量、二氧化硫排放量在“十一五”期间年均分别下降4%、4%、2.7%的基础上，继续有所下降。

　　——改革开放迈出新步伐。实际利用外资年均增长20%以上，进出口总额年均增长20%以上。到2012年，私营企业总数突破8万户，个私经济注册资本超过1500亿元。新增境内外上市企业50家，累计募集资金达到150亿元。政务、商务、社会管理信息化程度明显提升，形成全方位、高水平、深层次的大开放格局和更具活力、更符合国际规则的经济体制环境。

　　——人民生活水平实现新提高。居民财产性收入普遍增加，中等收入者占多数，城镇居民人均可支配收入、农民人均纯收入分别达到30000元、15000元以上。社会就业更加充分，五年累计增加城镇就业30万人以上，失业率控制在4.5%以内。实现主要社会保障全覆盖，形成功能完善、多层次、更普惠的保障体系。

　　——城乡建设展现新面貌。高速公路网络全部形成，建设京沪高铁、沪宁城际两条铁路常州段，完成飞机场改造升级。南部新城基本建成，北部新城初具规模，老城区经过改造呈现崭新面貌。建设城市快速高架环，快速公交网基本形成，奥体中心、文化中心、传媒中心等一批功能性建筑建成投运，所有菜市场全部改造提升。新运河建成景观航道，老运河经改造逐步成为城市文化和旅游品牌。城市绿化覆盖率、森林覆盖率分别达到45%、22%以上，绿化常州大地的目标基本实现。小康家园建设取得新进展，村容村貌呈现新气象。城市化率达到65%以上。建成国家生态市。

　　——和谐常州开创新局面。“五大建设”全面推进，各项事业协调发展，社会基本公共服务均等化程度显著提高，人民群众在政治、经济、文化等各方面的权益和社会公平正义得到保障，社会安定有序、和谐稳定，逐步实现学有所教、劳有所得、病有所医、老有所养、住有所居，全市人民安全感、幸福感进一步增强。围绕建设全国文明城市，努力把常州建成一个充满温馨和魅力的平安之城、慈善之城、活力之城、文明之城。

　　今后五年工作中必须牢牢把握以下五个重点：

　　（一）切实转变经济发展方式，推动产业结构优化升级，实现经济又好又快发展

　　实施先进制造业与现代服务业双轮驱动、融合发展，促进经济从粗放发展向集约发展、重点发展向优化发展转变。坚持以增量投入优化产业结构，以科技创新实现产业升级，以外向带动提升产业层次，以环保倒逼导向产业优化，以企业裂变发展促进产业集聚，使我市的产业层次有一个明显的提高。

　　（二）深化改革，扩大开放，进一步增强经济社会发展活力

　　着力推进政府职能转变，把更多的精力放到社会管理、公共服务和优化发展环境上来。健全现代企业制度，积极推动企业上市，加快培育一批具有国际竞争力的大企业集团。加快发展市场中介组织，健全和活跃土地、资本、技术、产权、人力资源等要素市场。优化外资结构，全面提升利用外资水平。加快“走出去”步伐，支持企业在研发、生产、销售等方面开展国际化经营。在更高层次上推进开发区建设，使各类园区成为我市科学发展的集聚地。

　　（三）加快城乡现代化建设，形成以工促农、以城带乡、城乡一体的互动发展格局

　　以城乡规划全覆盖为龙头，加快构建布局合理、功能互补的现代城镇体系。大力提升中心城区现代化水平，加快建设南北新城，形成三城联动、南北一体的城市新格局。加大交通城建基础设施投入，建设畅通便捷的立体交通网络，形成较为完善的城市综合功能服务体系。统筹城乡产业布局、基础设施建设和社会事业发展，着力发展现代高效农业，加快推进社会主义新农村建设。

　　（四）注重社会建设，着力保障和改善民生，促进经济社会协调发展

　　实施更为积极的就业政策，完善就业援助、技能培训和创业帮扶机制，以创业带动就业。促进社会保险、社会救助、社会福利、慈善事业相互衔接，进一步提高社会保障水平。推进区域教育现代化，实现各类教育均衡发展。加强公共卫生体系建设，构建城乡一体的卫生服务网络。努力促进文化事业大发展、大繁荣，全力办好十七届省运会。完善“大防控”、“大调解”机制，维护社会和谐稳定。

　　（五）落实环保优先，建设生态文明，构建资源节约型、环境友好型社会

　　通过大面积建绿、严标准治水、高起点生态修复、长效化城市管理、民本化居住保障，营造人与自然和谐相处的人居环境。坚持“四严三调”，实行严格环保准入门槛、严格环保执行标准、严格环保监管执法、严格环保考核评价，调整发展思路、调整产业结构、调整企业布局；推进“六治一绿”，治水、治气、治音、治山、治村、治废和全民造绿，实现环境水平新提升。全面建立节能减排硬性约束机制，积极发展循环经济，提高集约利用土地水平，增强可持续发展能力。

　　各位代表，未来的五年，既是我市巩固提高小康水平的重要时期，也是我们从全面小康向基本实现现代化迈进的关键时期。只要我们认准目标，坚定不移，锐意进取，开拓创新，就一定能把常州建成经济发达、科教先进、文化更加繁荣的城市；建成生活富裕、生态良好、人居环境更加优美的城市；建成风正气清、社会和谐、人民具有更高文明素质的城市；建成更加开放、充满活力、在长三角具有重要地位的城市。常州的明天一定会更加美好！

　　三、2008年主要工作建议

　　2008年全市主要预期目标是：

　　——地区生产总值增长17%以上；

　　——地方一般预算收入增长18%以上；

　　——全社会固定资产投资增长20%以上；

　　——社会消费品零售总额增长17%以上；

　　——注册外资实际到帐额增长20%以上；

　　——全社会研究与开发经费支出占地区生产总值的比重达到1.8%以上;

　　——城镇居民人均可支配收入增长13%以上；

　　——农民人均纯收入增长10%以上；

　　——城镇登记失业率控制在4%以内；

　　——万元地区生产总值综合能耗下降4.8%；

　　——化学需氧量排放削减率4%，二氧化硫排放削减率2.74%；

　　——环境质量综合指数达到83分以上。

　　为实现上述目标，按照“又好又快推进年”的总体要求，今年将重点抓好以下七项工作：

　　（一）突出科技创新，优化产业结构

　　着力提高科技创新能力。坚持市场导向、政府推动、企业主体，积极实施新一轮科技倍增计划。重点支持天合、常牵等一批科技领航型企业和100家市创新试点企业的科技创新，新增省级以上高新技术企业100家、企业“一站两中心”10家以上，力争实现企业科技投入75亿元。加快科教城建设，重点建设中科院常州中心等研发机构和大学科技园。切实加强各辖市区的科技创新和创业平台建设，逐步形成企业、区（市）和市三级科技创新平台联动的新格局。促进产学研联合，力争引进科研机构30家、科技成果300项，组织150项市级以上重点科技项目。实施“千名海外人才集聚工程”，引进海外归国人才300名，领军型创新创业人才20名，高层次研发人才50名。

　　做强做优“五大产业”。大力发展装备制造业，打造输变电设备、轨道交通设备、工程机械及车辆、新型农业机械、数控机床及基础装备五大产品群；大力发展电子信息产业，打造计算机及通讯设备、数字视听及电子元器件两大产品群；大力发展新能源及环保产业，打造太阳能光伏产品群，积极扶持风电产业的发展；大力发展新材料产业，打造高分子材料产品群；大力发展生物医药产业，打造生物工程及医药产品群。

　　大力发展现代服务业。加快凌家塘农副产品、横林地板等大型专业市场建设，重点发展武进保税物流中心等8个物流基地，积极打造城市中心商圈和特色商业街区。加快推进软件园和动漫基地建设，大力发展国际服务外包，重点抓好软件研发、信息服务、研发中心和动漫产业。加快春秋淹城、太湖湾、茅山旅游度假区等景区建设，加大旅游促销力度，力争接待国内外游客突破2000万人次，旅游总收入突破200亿元。积极推进金融改革创新试点工作，引进2家以上股份制商业银行或外资银行。

　　积极培育具有竞争力的大企业大集团。加快企业联合重组步伐，鼓励企业进入资本市场直接融资，积极开展国际经济合作，做优水平，做大规模，做强产业，努力形成一批在国内行业中有影响力和竞争力的大型骨干企业。年内新增营业收入超100亿元的企业集团1家以上、超50亿元2家以上，力争有8家以上企业上市。

　　继续加大有效投入。重点发展技术含量高、附加值高、低污染、低能耗的“两高两低”项目，不断提高投入产出强度。力争全社会固定资产投资达到1500亿元，其中工业有效投入800亿元。组织实施好172项市重点项目、150个市级重点工业项目。

　　（二）坚持量质并举，扩大对外开放

　　提升利用外资的总量和水平。找准我市产业与国际大公司产业转移的结合点，主攻大项目，新增总投资3000万美元以上的大项目20个，力争超5亿美元项目取得突破。继续做好“以外引外”，积极引导民营企业多形式、多渠道对外合作。充分发挥开发区招商引资主阵地的作用，开发区利用外资占全市的比重超过70%。

　　加快开发区转型升级。按照“集约、集聚、创新、生态”的思路，注重园区环境建设，高效集约利用土地，创新体制机制，扩大招商队伍，按照产业发展规划招商引资、招商选项、招才引智，实现开发园区由量的扩张向质的提高转变。继续加大园区基础设施投入，提高大项目的承载能力。

　　推进外贸外经健康发展。适应国家外贸政策，调整出口产品结构，扩大机电产品和高新技术产品出口份额，鼓励企业输出成套设备和技术。引导企业遵守国际贸易规则，支持和帮助企业应对反倾销和贸易壁垒。推进“走出去”战略，支持有条件的企业到境外设立贸易窗口和加工贸易企业，不断扩大外经市场份额。与此同时，进一步扩大对外交流，拓展友城工作。

　　（三）统筹城乡发展，壮大县域经济

　　着力发展现代高效农业。坚持以项目为抓手，加快发展高效型、生态型、质量品牌型和外向型农业，全市“三资”开发农业达到28亿元。继续壮大优质粮油、花卉苗木、现代畜牧、特种水产、时令水果等优势产业带，逐步形成3个5-10亿元产值的生产基地，设施农业、高效渔业和规模种植业面积分别增加1万亩、3万亩和6.5万亩。积极推进“一村一品”、“一镇一业”，重点抓好20个市级农业专业特色村建设。

　　有序推进农村“三集中”。加快镇村规划的编制，实现农村规划全覆盖，促进农业向规模经营集中、工业向园区集中、农民向城镇和农村新型社区集中。继续抓好8个市级现代化新农村建设示范镇、20个重点居住示范村和100个居住示范点建设。进一步深化农村各项改革，逐步扩大农业保险范围，引导农民组建土地股份合作社，年内符合农村集体经济股份合作制改革条件的村组全面完成改革任务。

　　进一步提升县区经济发展水平。金坛、溧阳要继续加快新型工业化步伐，重点发展太阳能光伏、输变电设备、机械装备等优势主导产业。武进、新北要优化产业结构，重点发展装备制造、新能源新材料、电子信息、软件动漫产业。天宁、钟楼、戚区要按照城市化的要求，大力发展现代服务业，加快向都市经济转型。

　　（四）优化城市建设，提升管理水平

　　加快综合交通体系建设。续建西绕城高速，开工建设常泰过江通道连接线，加快形成“三纵四横一环线”高速公路主骨架。实施338省道等6个改造项目，提升国省干线公路等级。全面完成第二轮农村公路建设任务，新建改建农村公路298公里。建成京杭运河改线航道配套工程和东、西港区，实施芜太运河、丹金溧漕河、锡溧漕河常州段三级航道整治工程，开工建设录安洲石化码头、夹江集疏运码头及配套工程。开工建设京沪高速铁路常州段，实施常州机场综合改造工程。

　　优化提升城市功能。按照“三城联动、南北一体”的要求，南部新城在全面建成5.6平方公里重点核心区的基础上，继续推进16.6平方公里的核心区建设，全面启动滆湖整治、保护和建设。北部新城要启动建设以京沪高铁常州站为中心的1.66平方公里的核心区建设。中心城区在完成高架路一期的基础上，新建改建中吴大道、飞龙中路、常新路、东方西路、丽华路、竹林北路延伸等11条骨架道路，打通晋陵南路、大仓路、红梅路等跨老运河通道，扩建龙游路、吊桥路、桃园路等10条城市次干道和支路。实施魏村水厂一期挖潜改造，开工建设二期工程。完成夹山垃圾填埋场续建工程，开工建设工业固体废弃物安全填埋场，生活垃圾焚烧发电厂竣工投运。实施城市防洪“大包围”工程，建成运北片北塘河、老澡港河、永汇河水利枢纽节点。

　　实施城乡绿化“十大工程”。（1）沪宁高速常州段两侧生态林工程；（2）太湖流域水系和内河河道生态绿化工程；（3）城区范围主要干道和道路节点绿化提升工程；（4）城市道口绿化工程；（5）城市公园增绿工程；（6）以18个乡镇为重点的集镇绿化工程；（7）以320个行政村为重点的村庄绿化工程；（8）企事业单位绿化工程；（9）围墙透绿工程；（10）垂直绿化工程。十大工程将新增绿地3000公顷以上。

　　深入推进城市长效管理。建立常州城市管理监督指挥中心，在城区范围内建设并运行数字化城市管理系统，将13项长效管理制度数字化，实现城市管理网格化、信息化、标准化。继续开展城市主要道路街景美化整治，重点实施局前街、怀德南路等8条道路和高架路两侧整治工程。开展“提升交通水平、方便市民出行”专项行动，建设畅通有序、安全文明的城市交通秩序。

　　（五）加强环境保护，建设生态城市

　　抓好太湖水污染治理。全面完成武进港、太滆运河、雅浦港三条入太湖河道及其主要支流的整治，确保水质长期稳定达标。继续开展工业污染源整治，年内关停并转化工企业363家。加强农业面源污染治理，实施生态修复和“三退三还”工程，重点推进太湖一级保护区生态修复。年内建成投运武南污水处理厂一期等一批污水处理工程。

　　完成节能减排任务。对化工、印染、冶金、造纸等重污染行业及城镇污水处理厂实施提标，完成已建成污水处理厂的提标改造。抓好重点企业的脱硫工程和年能耗3000吨以上标煤企业的节能工作，开展重点企业能源、清洁生产审计和项目节能评估审查，组织实施50项节能降耗和循环经济重点项目，对80家重点能耗单位实施节能监测。

　　确保环境综合质量全面达标。以水质达标为重点，加强对水环境功能区水质考核断面的监控，严厉处罚偷排、超排等违法行为。推进老城区20家污染企业的搬迁。开工建设江边污水处理厂二期工程，继续实施城区污水截流工程。启动饮用水源地及备用水源地的保护和建设。确保年内金坛、溧阳建成国家生态市，武进建成国家生态区。

　　（六）统筹协调发展，促进社会和谐

　　促进教育优质均衡发展。加快推进教育现代化工程，力争年内所有辖市区均实现区域教育现代化。积极实施素质教育，深化课程改革，加大对农村教育的扶持力度，进一步提高基础教育水平。加强在常高校能力建设，彰显职业教育特色，努力完善终身教育体系。加强教育基础设施建设，加快推进旅游商贸高职校、少年科学艺术宫三期和刘国钧高职校等项目。

　　增强地方文化发展活力。加大对历史文化街区及沿古运河历史文化带的保护和利用，逐步修复历史文化名园。大力发展文化事业和文化产业，大剧院年内建成开放，工人文化宫影剧院全面完成改造并对市民开放，启动建设现代传媒中心。整理地方典籍，创作文化精品，传承常州文脉。深入开展群众性精神文明创建活动，完善三级文化网络，丰富群众生活。

　　大力发展各项社会事业。完善覆盖城乡的公共卫生体系，着力提高社区卫生服务水平。建成市疾控中心、一院、三院门急诊大楼、中医院门诊病房楼，加快建设阳湖医院、新北医院。深入开展全民健身运动，办好市第十三届运动会，备战第十七届省运会，建成奥林匹克体育中心，举办好中国羽毛球大师赛等重大赛事。做好国防动员、民兵预备役、拥军优属和优抚安置工作，切实抓好外事、侨务、对台事务、地方志、民族宗教、防震减灾、关心下一代、人口与计划生育等工作，支持发展妇女儿童、老龄和残疾人事业。

　　完善社会保障体系。扩大社会保险覆盖面，提高社会保障能力，年内城镇养老、医疗、失业三大保险综合覆盖率稳定在97%以上。建立新型农村养老保险制度，逐步实现农民工工伤医疗保障。完善各类社会保险政策，逐步做实基本养老保险个人账户。

　　做好社会管理和安全生产工作。充分发挥社区和村自治组织的作用，加强流动人口管理与服务。健全安全生产监管体系，启动建设综合监管和重大危险源预警平台，全力做好事故减控，防止重特大安全事故的发生。健全应对各类突发性事件的工作机制，提高公共安全应急处置能力。开展食品药品安全示范镇和安全社区创建活动，提高食品药品安全保障水平。认真做好信访工作，妥善化解社会矛盾。依法惩处各类违法犯罪行为，切实维护社会稳定。

　　加强民主法制建设。深入推进“五五”普法，提高法律援助和服务水平。全面推进政府依法行政和政务公开，落实行政执法责任制。自觉接受市人大和市政协的监督，主动听取民主党派、工商联、无党派人士和各人民团体的意见和建议，认真办理人大代表、政协委员的议案、建议和提案。

　　（七）坚持发展富民，实现成果共享

　　以创业带动就业、促进增收。加大创业扶持力度，提高创业在就业中的比重。年内新增城镇就业7万人，援助困难人员就业3200人，实现再就业1.8万人。充分发展民营经济，放宽工商登记准入限制，加大政策扶持力度，扩大中小企业信用担保资金规模，完善风险投资体系。确保新增私营企业6000户以上、个体工商户1.5万户以上、注册资本80亿元以上。

　　继续为民办好十件实事。（1）建设“九公园三绿地”。建成西林、假日、恐龙园生态、飞龙公园，改建东坡、圩墩公园，启动建设东经120、华山、录安洲生态湿地公园，新建桃园绿地二期、金源绿地、武进汽车城绿地，实现所有公园免费开放。（2）“清水工程”。对潢河、通济河等9条城郊结合部河道进行整治，对白荡河、采菱港等19条河道进行清淤，续建北塘河城区段生态景观河道，确保城区水环境专项整治全面达标。（3）小康家园工程。对全市村庄实施“三清一绿”，清垃圾、清粪污、清河塘和村庄绿化；对100个村庄实施“五化三有”，道路硬化、村庄绿化、卫生洁化、河塘净化、环境美化和有公共服务中心、有长效管理机制、有乡村文化；建设10个更高标准的小康家园示范村。同时，对2个辖市、10个乡镇、170个行政村实施新农村电气化建设和改造。（4）公交惠民工程。BRT快速公交一号线全线开通；市区范围实现村村通公交；新增空调公交车600辆；建成区36条线路全部空调车运行。（5）改造升级菜市场。内部提升15个，原址开工改造3个，搬迁移建开工11个。（6）全面实施住房保障。新增廉租房1000户；新开工建设50万平方米经济适用房，年内提供2000套左右；公积金扩面5万人，发放低息贷款18亿元以上。（7）健全社会保障、救助体系。提高企业离退休人员退休金发放标准；实施新型农村养老保险制度，覆盖面达到50%；新型农村合作医疗市、辖市（区）财政补贴标准每人再提高20元，住院费用补偿率达35%以上；城乡低保月标准提高20元以上，年底增发一个月全额低保金，对低保边缘困难群众年底按户发放800元的一次性生活补助金；对低保、特困群众在市二级以上医疗机构就医的，实行“十免十减半”，同时对医疗费、大型医疗设备检查费等减免30%；继续按去年标准对三类重大疾病实行一次性救助，城市社区实现爱心慈善超市全覆盖。（8）城市社区卫生惠民服务。门诊实行“六免二减”，免一类疫苗接种、挂号、诊疗、健康建档、康复训练、计划生育指导等费用，减参保人员检查和治疗费用的10%；建立二级以上医疗机构与社区卫生服务机构结对帮扶、双向转诊绿色通道，逐步实现“小病在社区，大病进医院，康复回社区”。（9）改造提升公厕。改造提升131座，新建39座，取消所有公厕收费。（10）老城区重点改造工程。启动中吴大道两侧等重点地区改造；全面实施兰陵片区改造，新建建筑15万平方米以上；完成老城区剩余的100多条“背街小巷”道路整治；完成46万平方米老小区整治和老城区高层住宅二次供水改造。

　　四、切实加强政府自身建设

　　新一届政府肩负着实现基本现代化的历史使命，肩负着全市人民的希望和重托。要把这一希望和重托化为发奋工作的强大动力，从加强政府自身建设着手，转变政府职能，提高自身素质，把新一届政府建设成为“爱民、亲商、高效、廉洁”的服务型政府。

　　（一）爱民：就是要视人民为父母、群众为亲人，全心全意为他们谋利益。坚持以人为本的原则，把人民群众的呼声作为第一信号，把人民群众的需求作为第一选择，把人民群众的满意作为第一标准。切实关心人民群众特别是困难群体的利益，确保每个困难群众都能有饭吃、有衣穿、有房住，遇到困难有人帮。多办老百姓看得见、摸得着、得实惠的实事好事，让人民群众切实感受到发展成果就在我身上、就在我家里、就在我身边。

　　（二）亲商：就是要“支持企业天经地义，服务企业关爱有加”。产业发展靠企业，财源增加靠企业，社会就业靠企业，没有企业的发展，区域经济就会成为无本之木、无源之水。每个企业的发展都不容易，在成功的背后，他们承受了很多的艰辛和风险。要真心诚意地去关心、支持他们，依法保护他们的合法权益，调动激励企业家的创业热情。深入做好企业的服务工作，做到“无事不扰，有求必应”，急企业所急，想企业所想，把企业发展中遇到的难点作为服务的重点，千方百计帮助他们化解发展中的难题，为企业发展创造一个良好的外部环境。

　　（三）高效：就是要简化、明确、快速、创新。要简化办事程序，缩短办事时限，把复杂问题简单化。多讲群众听得懂的“地方话”，多做帮助企业排忧解难的简化式。对群众和企业的诉求要有明确的解决方案，对不符合条件的申请事项要一次性告知所缺条件。办事节奏要快，今日事今日毕，对群众和企业需要帮助解决的问题要“马上办”，并千方百计帮助他们“办上马”。要善于创新，不唯书，不唯上，只唯实，只要有利于常州的发展和全市人民的根本利益，就要敢想、敢闯、敢试，就要放胆去做。

　　（四）廉洁：就是要廉洁自律，既要干事，又要干净，要堂堂正正做人，清清白白做官，踏踏实实做事。全面落实廉政建设责任制，健全和完善惩治和预防腐败体系，努力从源头上预防和治理腐败。每个政府组成人员，都要身体力行，从我做起，以廉洁自律的实际行动，来赢得全市人民的信赖和支持。

　　各位代表，常州全面小康和现代化建设前程似锦、催人奋进。让我们在中共常州市委的领导下，万众一心，奋发图强，解放思想，开拓创新，向着率先基本实现现代化的宏伟目标奋勇前进！
